# Curability of Intestinal Perforation in Typhoid Fever

**Published:** 1855-04

**Authors:** 


### RECORD OF MEDICAL SCIENCE.

### Meeting of the College of Physicians.

*Curability of Intestinal Perforation in Typhoid Fever.*—Dr. Wood made the following remarks on this subject:—

The Fellows of the College may perhaps remember that, on two occasions, I have called their attention to cases of recovery from peritonitis in typhoid fever, occurring under circumstances which rendered the supposition highly probable that perforation of the bowels had taken place. A notice of the two cases was published in the *Transactions* of the College, to which I would refer. (See *Transactions*, N.S., vol. i. pages 32 and 418.) In relation to one of these cases, I have scarcely entertained a doubt as to the existence of perforation; and in the other, it appeared to me in the highest degree probable.

Subsequently, I have seen an account of two fatal cases of typhoid fever with sudden peritonitis, which occurred in the Charity Hospital Pàris, and in which, upon the most careful examination after death, no perforation could be discovered. A notice of these cases may be seen in the *Medical Examiner* (N.S., x. 120.) It cannot be denied that they tend to throw some doubt upon the reality of perforation, in the few cases which have been published of recovery from peritonitis in typhoid fever, supposed to have had this origin. But I have recently had under my care, in the Pennsylvania Hospital, a case of typhoid fever which, I think, affords satisfactory evidence of at least the possibility of recovery under the circumstances referred to; that is, when peritonitis has occurred in consequence of perforation of the bowels in that disease. To offer to the College a brief sketch of this case is the object of the present communication.

T. C., a young man, about 23 years old, was admitted into the Hospital on the 21st of November. His friends stated that, three weeks before his admission, he had been attacked with a chill, and had been unwell ever since. At the time of his entrance, he had all the characteristic symptoms of typhoid fever in its advanced stage; among them, a pulse of 128 in the minute and very feeble, a perfectly dry tongue, muttering delirium, diarrhœa, and tympanitic abdomen with the rosecolored spots and tenderness on pressure. There was also cough, with embarrassed respiration and evidences of pulmonary congestion. He was put on the use of oil of turpentine, and small doses of the blue mass, opium, and ipecacuanha, with emollient poultices to his abdomen, dry cups to his chest, and wine-whey, soup, and milk, to support his strength. On the 24th, his tongue had become moist, his abdomen less tumid and tender, and his pulse reduced to 115. So far as the fever was concerned, he seemed on the way to convalescence; but the pectoral symptoms had increased, and pneumonia was decidedly developed. The remedies were continued, with the addition of a large blister to his chest. But the pulmonic affection rapidly gained ground; the respiration was very much oppressed; the skin became cold and clammy, and the pulse extremely feeble; and life was sustained only by the administration of the most active stimulants. He died on the first of December, obviously of the disease of his lungs, the abdominal symptoms having almost entirely disappeared.

On examination after death, the upper half of the right lung was found in a complete state of gray hepatization, being everywhere infiltrated with pus, and so soft that the handle of the knife could be passed through it in all directions with very little resistance. The remainder of the right lung, and most of the left, were more or less congested. There could be no doubt as to the cause of his death. I was not present at the examination of the body; and, though the lungs and intestines were kept for my inspection, I had no opportunity of seeing the bowels in *situ*. Dr. Forbes, however, one of the resident physicians of the hospital, who made the autopsy, was kind enough to present me with the following account, in substance, of what he discovered on opening the abdomen.

Evidences of previously existing peritonitis were exhibited in small portions of semi-organized coagulable lymph adhering to the bowel and abdominal parietes, chiefly in the right iliac region, where, at one spot, it served to agglutinate the small intestine to the anterior wall of the abdomen. There was little or no liquid in the cavity, and no fecal matter. The adhesion was carefully separated by the thumb-nail, when a portion of the contents of the bowel escaped into the abdomen at the point of separation. On further examination, a perforation of the intestine was found in the middle of an ulcer, which appeared to be healing at its edges, as were several other ulcerated surfaces.

When exhibited to myself, the intestines were, as already stated, separated from the body, and had been laid open. A considerable number of healing ulcerated surfaces were visible along the ileum, in the situation of the aggregated glands; and, in the middle of one of these ulcers, near the ileo-cœcal valve, was an oval opening, about half an inch in length, quite through the bowel, with a smooth, rounded edge, which had certainly not been produced either by tearing or by the knife. There could not be the least doubt that it was a perforating ulcer. Near its edge, on the peritoneal surface of the bowel, was an adhering layer of semi-organized coagulable lymph, about an inch and a half in diameter, and here and there smaller patches, with some shreds adhering somewhat loosely to the bowel, at a little distance from the surface of adhesion, showing that the inflammation had extended some distance beyond the outer limits of that surface.

The College will, I think, agree with me in the conclusion that here had been a case of peritonitis from perforation, though of no great extent. Before any considerable portion of the intestinal fluid had escaped, adhesion had taken place around the opening by means of the exuded fibrin, and further mischief was thus prevented. The inflammation had probably begun to subside before the patient entered the house; and the 24th may be considered as the commencement of convalescence, so far as the peritonitis was concerned. I presume that the patient would have recovered, but for the supervention of pneumonia, which, in consequence probably of the general debility and bad state of the blood, passed very rapidly into the third stage, or that of suppurative disorganization.

If my interpretation of this case is correct, it proves pretty conclusively the possibility of a cure of peritonitis from perforation in typhoid fever, and supports, if it does not justify, the views taken of the pathology of the first two reported cases.—*Trans. of the College of Phys. of Philad. N. S. Vol. ii., No. 7.*

*Meeting of the College, Feb.* 7, 1855.

*Peritonitis in Typhoid Fever, without Intestinal Perforation.*—Dr. Wood made the following remarks on this subject:—

"Since my last communication to the College, a case has occurred to me at the Pennsylvania Hospital, which has some bearing on the question referred to in that communication; whether, namely, recovery has ever really taken place from peritonitis resulting from intestinal perforation in typhoid fever. What I stated at a former meeting of the College proves, I think, the possibility of such an event. The case of which I am about to give an outline shows, like those reported as having occurred in one of the hospitals at Paris, that we can never be positively certain, in any particular instance of peritonitis in the advanced stage of typhoid fever, that it originated in perforation.

"J——— B———, aged seventeen years, entered the hospital on the 2d of January. According to his own account, he had been three days ill; but the symptoms, which were those of typhoid fever in the middle of its course, evinced that he was in the second week of the disease. The tongue was quite dry, and the abdomen tympanitic; and the characteristic red spots, if not present at the time of his entrance, made their appearance very soon afterwards. Under the plan of treatment usually pursued in the hospital, including the use of oil of turpentine as an alterative to the ulcerated surfaces in the intestinal mucous membrane, he gradually amended; and on the 20th, I presented him to the class in attendance upon the clinical lectures as quite convalescent. On the 21st, however, he was suddenly seized with severe abdominal pains, tenderness on pressure, and great prostration; and it was evident that he was laboring under an attack of violent peritonitis. I had no doubt of the existence of perforation of the bowel, which I felt disposed to ascribe to an orange which he had surreptitiously eaten a few days previously, and of which, in order to conceal his violation of the rules of the ward, he had probably swallowed the seed and rind. With this conviction of the nature of the case, I directed the use of sulphate of morphia largely, according to the well-known plan of Drs. Graves and Stokes, with perfect rest, a blister over the abdomen, and, towards the close of the case, stimulants in order to support life. The system never fully reacted, and death took place on the 25th.

"Dr. Forbes, one of the resident physicians of the hospital, opened the body, and found a small quantity of turbid liquid in the abdominal cavity, with evidences of almost universal inflammation of the peritoneum; but, on the closest and most careful inspection, could discover no perforation of the bowel; nor was there the slightest fecal odor in the liquid effused. I had afterwards an opportunity of examining the whole of the intestines, though not *in situ.* The internal surface of the ileum presented several recent and well-defined *cicatrices,* finely marking the boundary of the patches of Peyer's glands, which I exhibited to the class as the finest example I had seen of this appearance; but there was not a single remaining ulcer visible, and I could find no sign of an opening. The peritoneal coating was covered with a very thin, glutinous, and translucent exudation of coagulable lymph, but with little redness. At no one point upon the surface were there more decided marks of inflammation than elsewhere, to indicate the possible seat of an opening. I was compelled, therefore, to regard the case as one of peritonitis without perforation. No discoverable cause of the affection existed; and I am inclined to rank this with those not very uncommon cases, in which peritonitis comes on without any special known cause, at the close of long-continued and exhausting affections, perhaps in consequence of depravation of the blood.

"This case is calculated to throw great doubt upon the existence of intestinal perforation in those instances of peritonitis, occurring in the advanced stage of typhoid fever, in which cures have been effected, of which I have been so fortunate as to witness two, in my own experience. They may have been, as in the case just related, nothing more than simple peritonitis without any opening whatever through the coats of the bowel. One important practical inference, however, may be deduced from them, viz: that the opiate treatment is the one best adapted to peritonitis occurring under these circumstances, whether with or without perforation; as several instances of recovery have taken place under that treatment, while I am not aware that one is on record effected under any other plan.—*Ibid.*

